# Construction of Recombinant Baculoviruses Expressing Infectious Bursal Disease Virus Main Protective Antigen and Their Immune Effects on Chickens

**DOI:** 10.1371/journal.pone.0132993

**Published:** 2015-07-13

**Authors:** Jingping Ge, Qi An, Shanshan Song, Dongni Gao, Wenxiang Ping

**Affiliations:** Key Laboratory of Microbiology, College of Life Science, Heilongjiang University, Harbin 150080, China; Thomas Jefferson University, UNITED STATES

## Abstract

In order to overcome the limitations of conventional vaccines for infectious bursal disease virus (IBDV), we constructed recombinant dual expression system baculoviruses with VP2 and VP2/4/3, the main protective antigens of IBDV. We compared the immune effects of the baculoviruses in avian cells and detected their control effects on chickens with infectious bursal disease. We used Western blot analysis to measure VP2 protein and VP2/4/3 polyprotein expression in avian cells infected using the Bac-to-Bac baculovirus expression system. The recombinant baculoviruses were used to vaccinate specific pathogen-free chickens, which produced specific protective antibodies and strong cellular immune responses. The results of the virus challenge experiment revealed that the protective efficiency of VP2 and VP2/4/3 virus vaccines were 95.8% and 100%, respectively, both of which were higher than the vaccine group (87.5%), and significantly higher than the control group (50%). The results demonstrated that the immune effect of BV-S-ITRs-VP2/4/3 was superior to that of BV-S-ITRs-VP2. Compared with traditional attenuated vaccine and genetically engineered live vector vaccine, the dual expression viral vector vaccine has good bio-safety. The results of this study provide a foundation for the further development of poultry vaccines, in addition to providing a useful reference for developing non-replicating live vaccines against other viral diseases.

## Introduction

Infectious bursal disease is a poultry disease caused by the infectious bursal disease virus (IBDV) [1]. Chickens infected with IBDV exhibit bursal atrophy and eventually die, causing a substantial economic loss for the poultry industry [2]. Vaccination against IBDV is currently considered as a viable option. Both inactivated and live vaccines are the most commonly used vaccines, but they each have disadvantages [3]. For instance, the immunization process of inactivated vaccines is time-consuming and laborious, requires a higher injection dosage [4]. Whereas, the attenuated live vaccine can only elicit a small amount of antibodies and fails to provide enough protection to chickens [5]. Therefore, there is currently a great research effort underway to find novel vaccines.

Compared with other expression systems, the baculovirus expression system has distinct advantages. It is capable of accommodating large fragments of exogenous genes [6], and modifying the post-translational products, without causing cytotoxic effects to cells [7]. Additionally, multiple genes can be simultaneously expressed by the baculovirus at high levels and the expression products can be conferred with biological function [8, 9].

VP2 is the main protective antigen of IBDV, which is involved in inducing virus neutralizing antibodies, cell apoptosis and antigenic variation [10, 11, 12]. The VP2/4/3 polyprotein can be exactly cut into the natural configuration of the VP2 protein, although the expression level is low [13]. Therefore, choosing the appropriate target gene is crucial.

In order to improve the efficiency of expression of the foreign genes mediated by the baculovirus in the host cell, researchers have attempted to change the type of promoter (e.g., Simian Virus 40 promoter, Cytomegalovirus CMV promoter, CMV early enhancer and chicken β actin promoter), and added appropriate regulatory expression elements to improve the efficiency of target gene expression. The CMV promoter is recognized as a strong promoter of the eukaryotic expression vector as it can regulates the expression of recombinant baculovirus in mammalian cells, in addition to driving foreign gene expression efficiently in poultry cells [14]. The display of vesicular stoma titis virus glycoprotain (VSV-G) on the recombinant baculovirus surface can increase the transduction efficiency of baculovirus in vitro and in vivo and significantly increase the cell tropism of baculovirus [15]. Furthermore, the woodchuck hepatitis virus post-transcriptional regulatory element (WPRE) and adeno-associated virus inverted terminal repeats (ITRs) also play vital roles in improving the expression efficiency of target gene and extending the expression time. Research has shown that inserting WPRE in the 3'UTR region of the target gene can increase the transfection efficiency of the exogenous gene 10-fold, without causing any cytotoxicity [16]. Furthermore, adding adeno-associated virus inverted repeats on both sides of the promoter expression cassettes causes the target gene to be continuously expressed at a high level.

In this study, different regulatory elements such as the CMV promoter, VSV-G, WPRE and ITRs were used to modify the dual baculovirus expression system to realize the expression of *vp2* and *vp2/4/3* genes of chicken IBDV. Using the baculovirus to directly infect poultry cells to prepare poultry vaccines is a foundation for future molecular immunology studies and research into generating an efficient genetically engineered vaccine.

## Materials and Methods

### Ethics Statement

Care of laboratory animals and animal experimentation were performed in accordance with animal ethics guidelines and approved protocols. All animal studies were approved by the Animal Ethics Committee of Harbin Veterinary Research Institute of the Chinese Academy of Agricultural Sciences (CAAS) and the Animal Ethics Committee of Heilongjiang Province (SYXK (H) 2006–032).

### Virus, plasmids and cells

IBDV virulent strain BC6/85 (CVCC AV7) was purchased from China Veterinary Microbiology Culture Collection. Plasmids pTZF-VP2, pA-8 and pS-CMV were previously prepared by the laboratory. The chicken embryo fibroblast cells and *Sf*9 insect cells were prepared by the laboratory.

### Construction and screening of recombinant baculovirus

Using pTZF-VP2 and pA-8 as templates, we amplified *vp2* and *vp2/4/3* target genes with two pairs of specific primers: Forward primer for VP2: 5’-GCGGGC-CCATGACAAACCTGCAAGATCAAAC-3’ (an *Apa* I site was introduced); reverse primer for VP2: 5’-GCGGTACCTCA*ATGATGATGATGATG-ATG*TTACCTTATGGCCG- TTAT-3’ (a *Kpn* I site was introduced); forward primer for VP2/4/3: 5’-GCGGTACCATGACAAACCTGCAAGATCAAAC-3’(a *Kpn* I site was introduced); and the reverse primer for VP2/4/3: 5’-GCGGTACCTCA*ATGAT GATGATGATGATG*TCACTCAAGGT- CATCAGAGAC-3’ (a *Kpn* I site was introduced).

**Fig 1:**
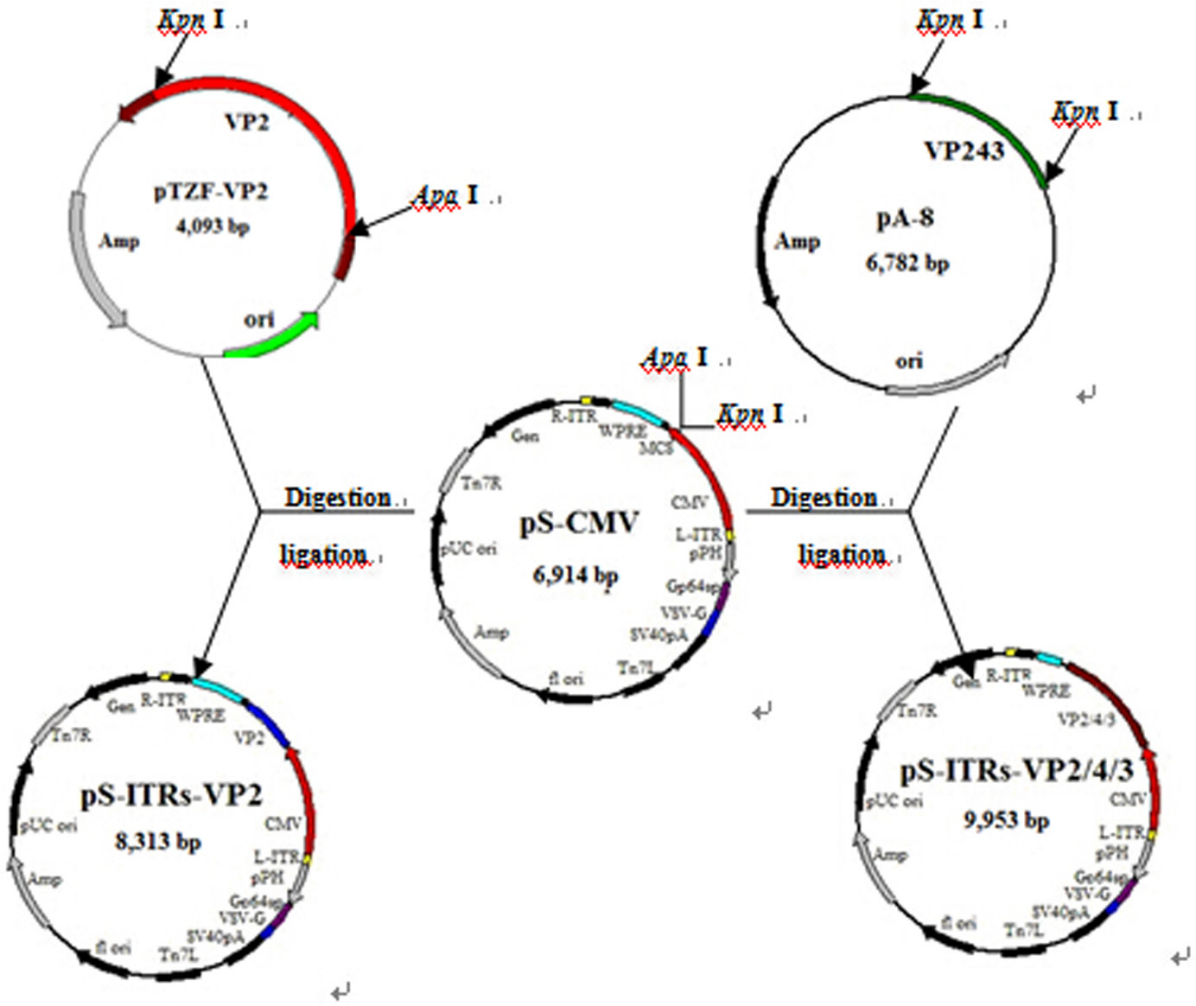
Construction of the baculovirus transfer vectors pS-ITRs-VP2 and pS-ITRs-VP2/4/3. Plasmids pTZF-VP2 and pA-8 were used as templates, *vp2* and *vp2/4/3* target genes were amplified and separately inserted into vector pS-CMV according to the respective restriction sites *Apa* I/*Kpn* I to construct the baculovirus transfer vectors pS-ITRs-VP2 and pS-ITRs-VP2/4/3.

The PCR products were recovered by 1% agarose gel electrophoresis. The target fragments of *vp2* and *vp2/4/3* were separately inserted into the vector pS-CMV according to their respective restriction sites *Apa* I/*Kpn* I and *Kpn* I/*Kpn* I. The positive recombinant plasmids, which were confirmed by PCR analysis and restriction enzyme digestion, were named pS-ITRs-VP2 and pS-ITRs-VP2/4/3 (Fig 1). As a result of the enzyme digestion, we obtained the backbone vector of 6.9 kb, VP2 fragment of 1.4 kb and VP2/4/3 fragment of 3.0 kb, as expected. The sequence analysis showed that the *vp2* gene was 1,399 bp, and the *vp2/4/3* gene was 3,039bp.

The *E*.*coli* DH10 Bac competent cells were prepared by the SEM method and plasmids pS-ITRs-VP2 and pS-ITRs-VP2/4/3 were transformed into *E*.*coli* DH10 Bac competent cells. The positive colonies were obtained by blue-white screening and the Bacmid DNA was extracted using the alkaline lysis method. The recombinants were confirmed as rBac-S-ITRs-VP2 and rBac-S-ITRs-VP2/4/3 by PCR amplification with M13 primer.

### Generation and purification of recombinant baculovirus

The baculovirus transfer vectors rBac-S-ITRs-VP2 and rBac-S-ITRs-VP2/4/3 were separately transfected into *Sf*9 insect cells, according to the liposome-transfection method. After incubation at 27°C for 5 h, 2 mL Sf900 II complete medium was added. The co-transfected supernatants were collected after 72 h of culture. The viruses were purified by plaque assays and then infected into *Sf*9 insect cells with several rounds to obtain higher titers of viral stocks. The virus titers calculated by plaque assay were 8.0 × 10^8^ (BV-S-ITRs-VP2) and 9.0 × 10^8^ (BV-S-ITRs-VP2/4/3). In addition, the viral genome was extracted and amplified in accordance with the primers, to confirm that the target genes were inserted into the recombinant baculovirus correctly.

### Expression of VP2 protein and VP2/4/3 polyprotein in chicken embryo fibroblast cells

The recombinant baculoviruses BV-S-ITRs-VP2 and BV-S-ITRs-VP2/4/3 at a multiplicity of infection (MOI) of 100 were separately added to the chicken embryo fibroblast cells at a density of 5 × 10^5^ cells/mL. Subsequently, sodium butyrate was added to a final concentration of 10 mmol/L. The virus infection experiment was carried out at 37°C with 5% CO_2_. The Dulbecco’s modified Eagle medium (DMEM) complete medium was replaced after 12 h. After 48 h, cells were lysed in lysis buffer and proteins were harvested and frozen at −20°C for short-term preservation.

### Sodium dodecyl sulfate-polyacrylamide gel electrophoresis (SDS-PAGE) and Western blot analysis

The purified proteins were separated by 12% SDS-PAGE electrophoresis and then transferred onto nitrocellulose membranes for Western blot analysis. The IBDV antiserum was extracted from chickens suffering from infectious bursal disease and diluted 1:1,000 (kindly provided by Harbin Veterinary Research Institute of Chinese Agricultural Sciences Academy). The horse radish peroxidase (HRP)-labeled rabbit anti-chicken IgG (purchased from Promega) was diluted 1:5,000 and was used to detect the expression of VP2.

### Chicken immunization

Fourteen-day old specific pathogen free (SPF) chickens were raised at randomly divided into six groups (A–E) with eight chickens in each group. The chickens in groups A, B and C were immunized with BV-S-ITRs-VP2, BV-S-ITRs-VP2/4/3 and BV-S, respectively. The chickens in group D were immunized with commercial vaccines. Chickens in group E were set aside for the challenged control. Chickens in groups A, B and C were injected with 10^9^ pfu recombinant baculovirus per chicken; And chickens in group D were immunized with 0.2 mL vaccine per chicken. Chickens of all the vaccinated groups were boosted with the corresponding vaccines at the same dosage 14 days after the first immunization. Blood samples were obtained from five immunized chickens selected at random from each group after 14, 21, 28, 35 and 42 days. The blood was separated by centrifugation, and the serum was collected for analysis. The serum was stored at −20°C in preparation for the enzyme-linked immunosorbent assay (ELISA) and the serum neutralization antibody titer determination.

### ELISA for detection of anti-VP2 antibodies

The serum antibody levels of the immunized chickens were detected using an ELISA kit (CK-E91977C, Baosight Company). In accordance with the instructions of the IBD antibody detection kit, the negative control, positive control and samples were added to the lath respectively, and 100 μL HRP-labeled detection antibody was then added to each well. After 1 h incubation at 37°C, 50 μL substrate A and substrate B were added, and then samples were incubated in the dark at 37°C for 15 min. Finally, 50 μL of stop solution was added and the OD value was measured at a wavelength of 450 nm.

### Virus neutralization titer assay

The serum neutralization test was performed to detect the neutralization of antibodies in vitro. First, the chicken embryo fibroblast (CEF) cells were prepared by our laboratory in advance. Then the test serum was incubated at 56°C for 30 min to inactivate the complement. The serum, diluted with PBS, was mixed separately with 100 TCID_500_ virus suspension and incubated at 37°C with 5% CO_2_ for 1 h. The mixtures were then transferred to 96-well plates with more than 80% confluent monolayers of CEF cells in DMEM complete medium containing 10% serum. The lesions of cells were observed and recorded every 12 h until 96 h. According to the Reed-Muench method [17], the median protective doseapp:addword:median protective dose (PD_50_) of each serum sample was calculated, in addition to the geometric mean titers (GMT) in each group, and the neutralizing antibody titer of the blood serum.

### Challenge with virulent IBDV

The chickens were challenged with virulent IBDV strain BC6/85, at 42 days post-immunization, via eye dropping with 100 median embryo lethal doses (ELD50) per chicken. The chickens were observed and any changes in feed intake, water intake, excrement and mental condition were recorded. All the challenged chickens were slaughtered at 5 days post-challenge. In our research, euthanasia was adopted in the experiment to minimize suffering of chickens. Once the chickens emerged signs such as loss of appetite and depressed, euthanasia was adopted in the experiment. Sodium pentobarbital solution was injected via vein intravenous with 80mg/kg, chickens would humanely sacrificed with less suffering. The chickens were dissected and the bursa-weight/body-weight ratio was calculated. Finally, the bursal lesions were observed and the clinical protection rate was calculated.

## Results

### Expression of VP2 protein

**Fig 2:**
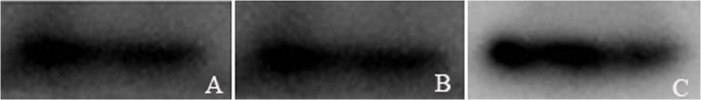
Western blot analysis to confirm VP2 expression. Chicken embryo fibroblast cells were infected with recombinant baculoviruses and cell lysates were used in Western blot analysis to detect VP2 protein expression. (A) BV-S-ITRs-VP2; (B) BV-S-ITRs-VP2/4/3; (C) *β*-actin control.

**Fig 3:**
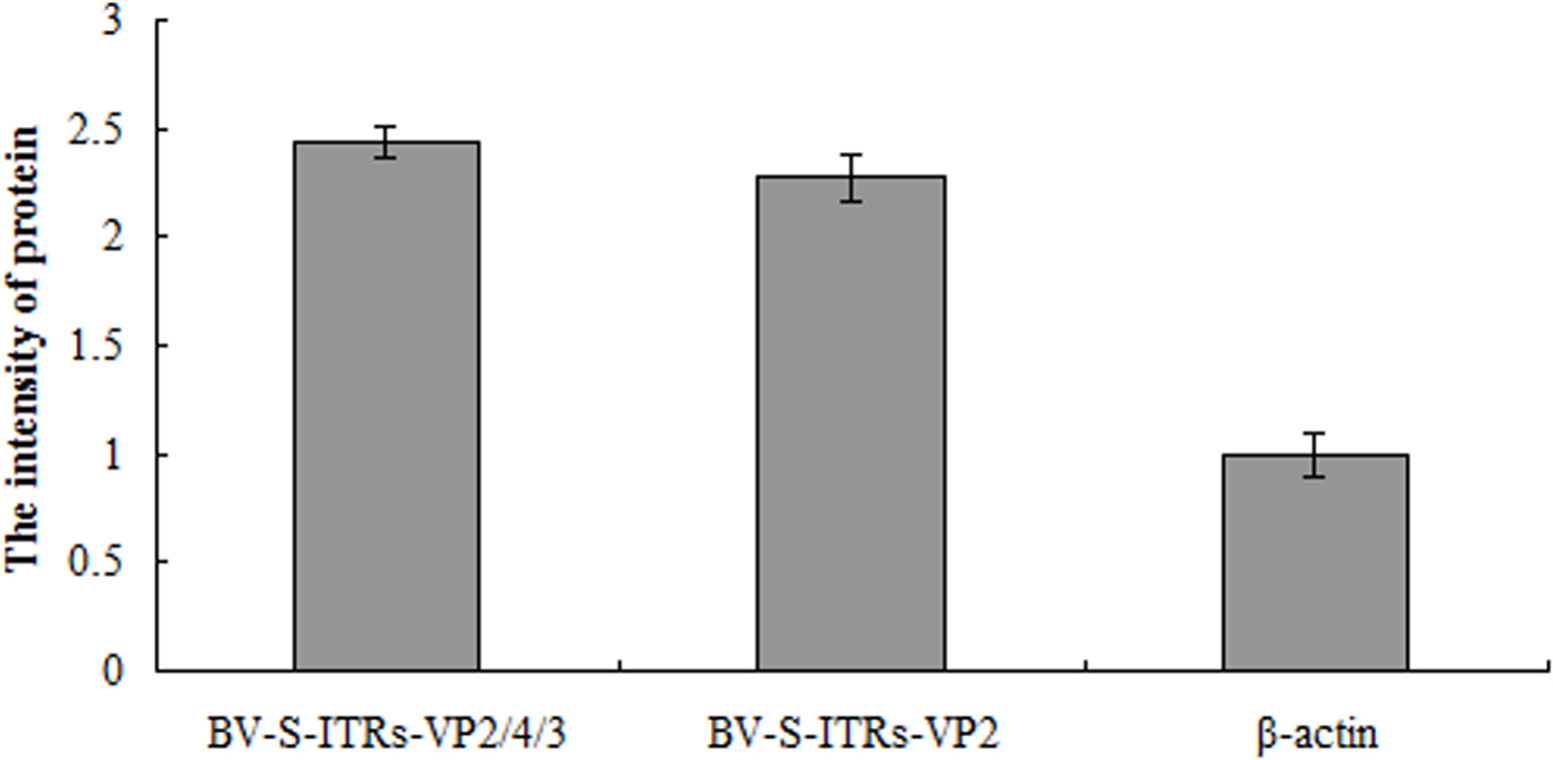
The relative expressed intensity of VP2 protein. (A) BV-S-ITRs-VP2; (B) BV-S-ITRs-VP2/4/3; (C) β-actin control. The VP2 protein intensity of recombinant baculovirus BV-VP2 and BV-VP2/4/3 was analyzed and the relative amount of VP2 protein was quantified.

The recombinant baculoviruses (BV-S-ITRs-VP2 and BV-S-ITRs-VP2/4/3) were used to infect the chicken embryo fibroblast cells, and VP2 levels in the cell lysates were detected by SDS-PAGE and Western blot analysis. As a result, a band was detected at a molecular weight of approximately 42 kDa, and no band was observed in the control (β-actin), which confirmed successful expression of the target antigenic protein VP2 (Fig 2). The intensity of protein expression was quantified by analysis software (Fig 3), which showed that the protein intensity of BV-S-ITRs-VP2 was 1.09 times that of BV-S-ITRs-VP2/4/3.

### Detecting VP2 antibody in chickens

**Fig 4:**
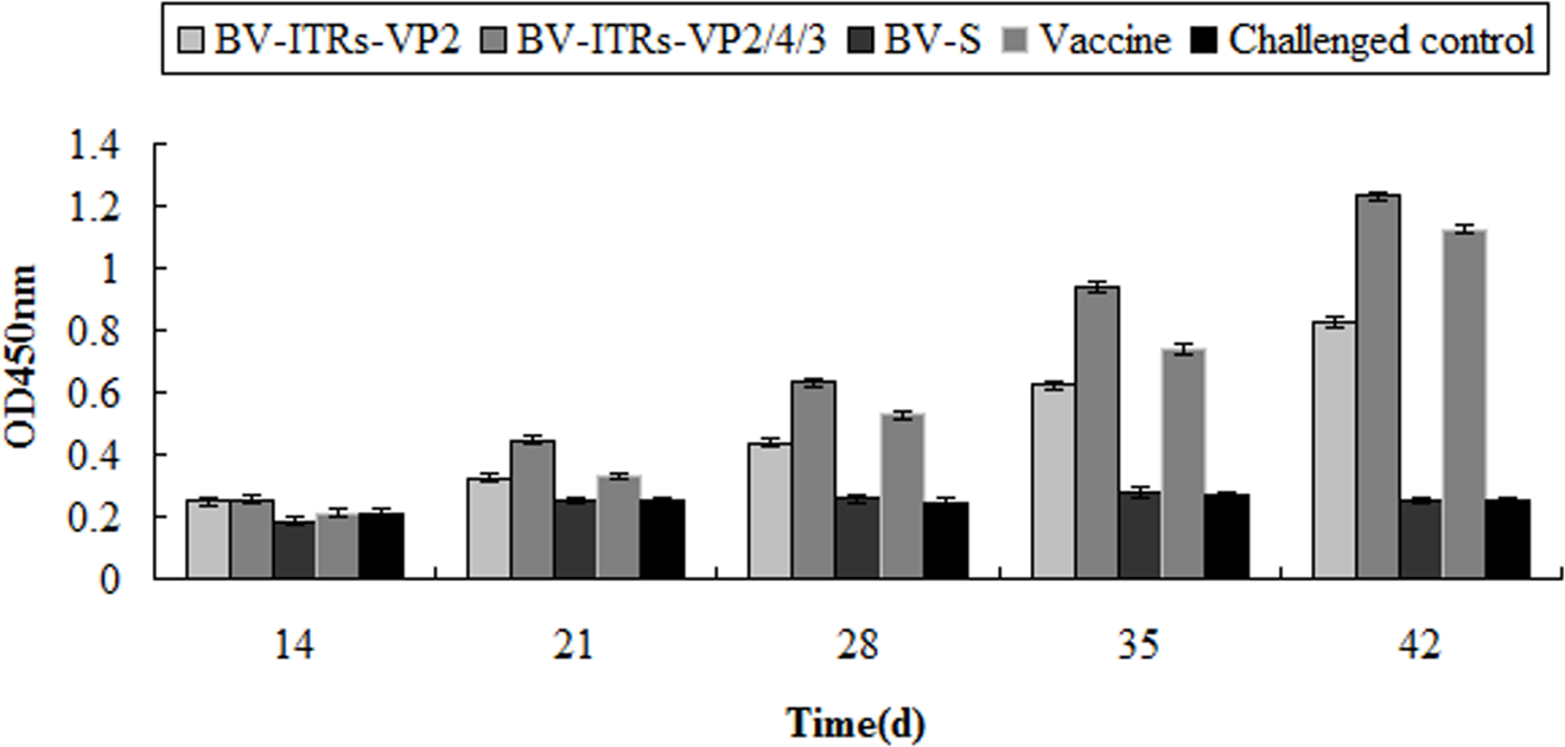
Antibody levels produced by chickens in each group. Chickens were immunized with corresponding vaccines at a dose of 0.2 mL per chicken and boosted with the same dosage 14 days after the first immunization. BV-S is an empty (control) vector without the target gene. The immune serum of chickens in each group was collected at 14, 21, 28, 35 and 42 days for the detection of antibody levels by ELISA.

**Fig 5:**
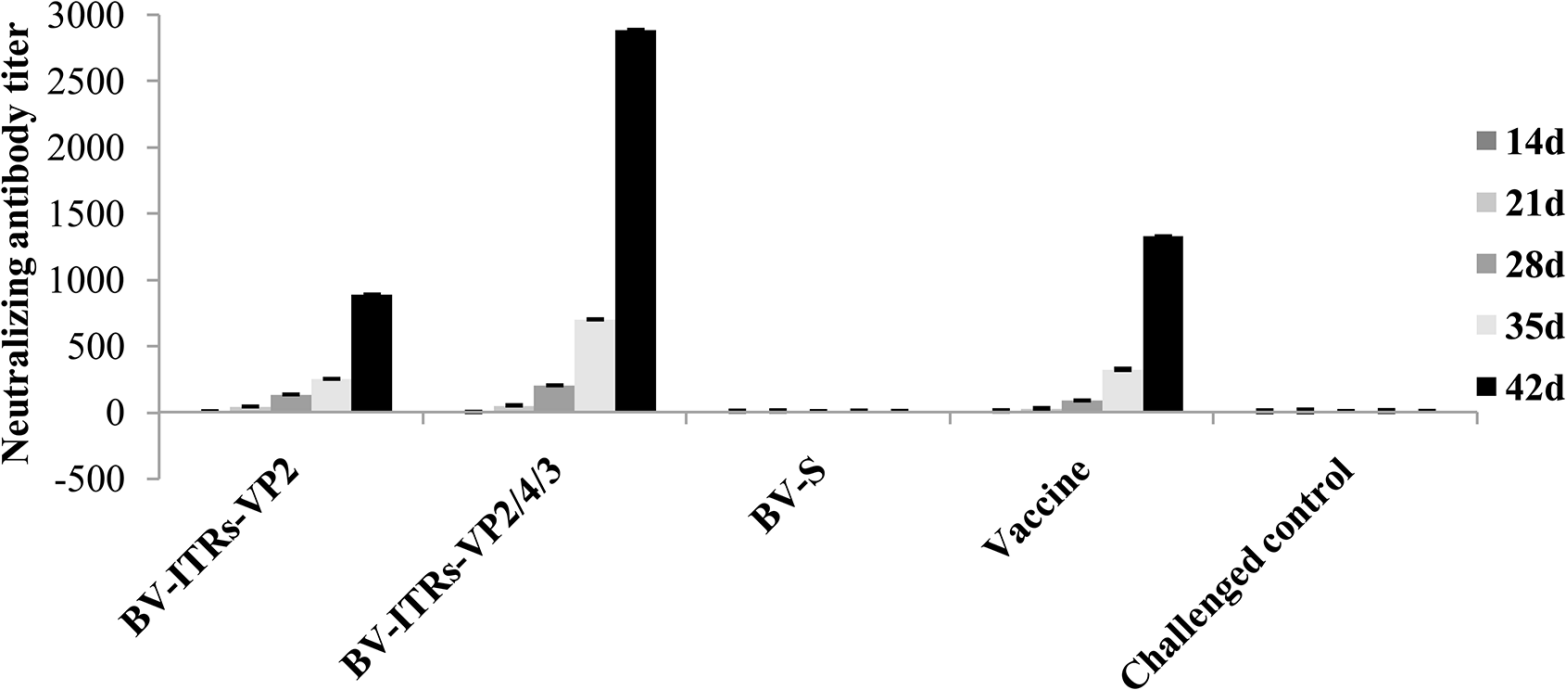
Comparison of neutralizing antibody titer induced by chickens in each group. Chickens were immunized with BV-S-ITRs-VP2/4/3, BV-S-ITRs-VP2, control plasmid BV-S and vaccine, respctively. Blood samples were obtained from immunized chickens selected at random from each group after 14, 21, 28, 35 and 42 days. The neutralizing antibody titers of the blood serum (GMT) of five chickens from each group were calculated.

ELISA was employed to detect the antibody levels of the immune serum at different times. The antibody levels of chickens increased gradually with the vaccination time, and peaked at 42 days post-vaccination. In the 42 days of vaccination, the antibody levels of the BV-S-ITRs-VP2/4/3 vaccine group reached 1.223, which was higher than that of the BV-S-ITRs-VP2 vaccine group of 0.824 and significantly higher than the control group of 0.246 (*P* < 0.05) (Fig 4). Therefore, the neutralizing antibody level induced by the VP2/4/3 vaccine group was higher than that induced by the VP2 vaccine group (Fig 5).

### Clinical observations and protection rates

After the first immunization and the booster immunization, no clinical responses were observed in the chickens from each group. After challenge with the virulent IBDV strain, chickens in groups C and E appeared depressed, had reduced feed intake and produced white stools. Four chickens from each group died 3 days post-challenge. One chicken in group D and one in group A suffered from an unidentified ailment and eventually died. There were no obvious clinical symptoms in the chickens in group B.

Five days after challenge, the protection rate of BV-ITRs-VP2/4/3 and BV-ITRs-VP2 were 100% and 95.8%, respectively, both of which were higher than the control group (50%), indicating that the vaccine group induced adequate protection against the virulent IBDV strains (Table 1).

**Table 1:** Clinical features and mortalities of experimental chickens.

Group	Vaccinated groups	Injected dose	Mean value of BBIX	Mortality
A	BV-S-ITRs-VP2	200 μL	3.45 ± 0.14	4.2%
B	BV-S-ITRs-VP2/4/3	200 μL	4.07 ± 0.19	0
C	BV-S	200 μL	3.03 ± 0.18	50%
D	Vaccine group	200 μL	3.13 ± 0.27	12.5%

The experimental chickens in groups A, B and C were immunized with BV-S-ITRs-VP2/4/3, BV-S-ITRs-VP2 and control plasmid BV-S at 10^9^pfu, respectively. The poison attack dose was determined as 100 ELD_50_ and the clinical features of chickens were observed and recorded. BBIX, bursa-weight/body-weight index.

### Anatomical study of atrophic bursa

**Fig 6:**
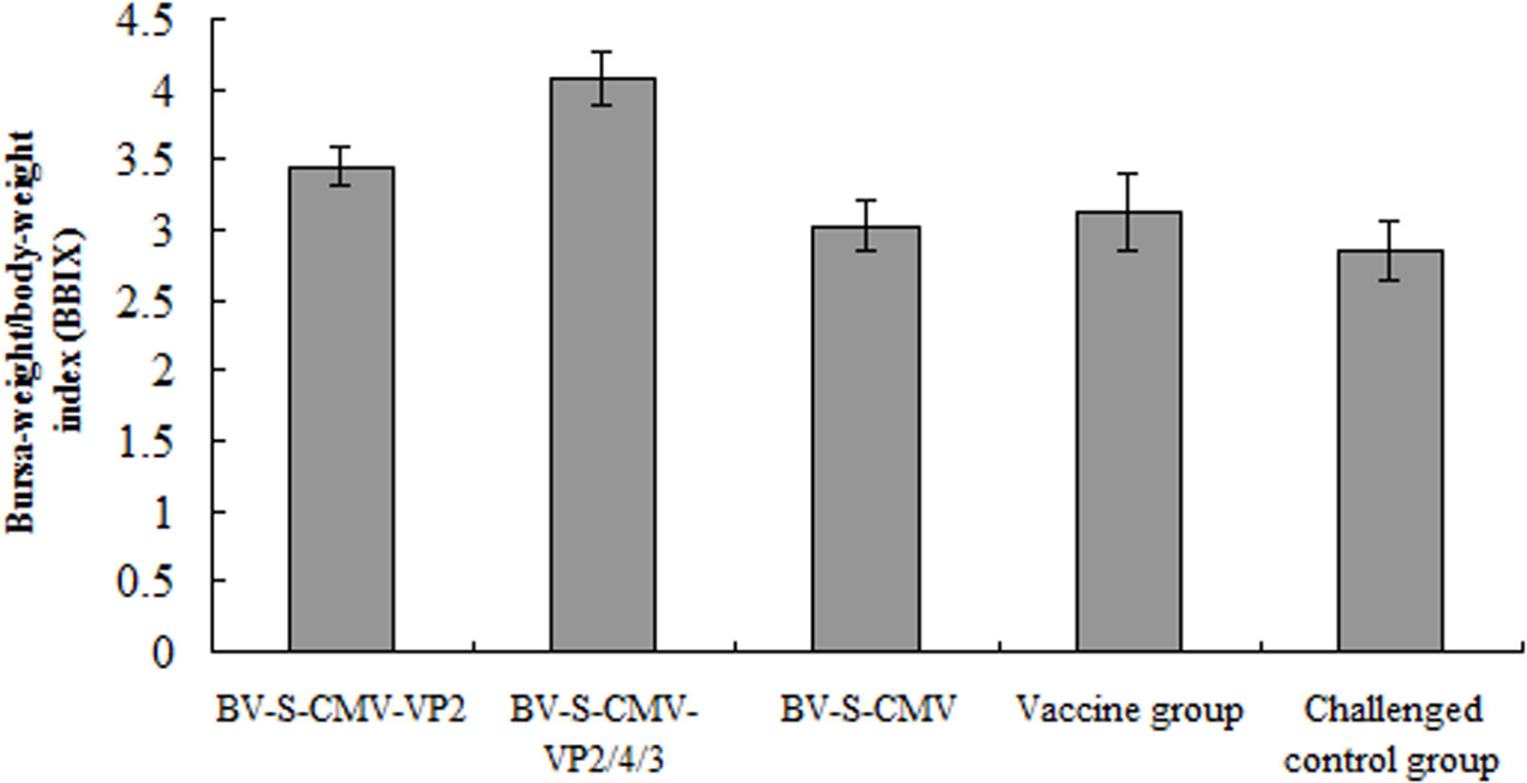
The mean bursa-weight/body-weight index (BBIX). Challenge was carried out via eye dropping with 100 ELD_50_ per chicken at 42 days post-immunization. The bursa-weight/body-weight ratio was calculated to assess the immune protection conferred by chickens in each experimental group.

Anatomical analysis revealed that the bursa of fabricius in most chickens from groups C and E were atrophic. There was one chicken from group D appeared to have atrophic bursa. There were three chickens in group A and two in group B with atrophy of the bursa. In the anatomy of the bursa we found visible necrosis and bleeding points in chickens from groups C and E, whereas, no similar lesions were found in the chickens from groups A and B. There were two chickens with severe bursal atrophy in group D. The mean value of bursa-weight/body-weight index (BBIX) of chickens was calculated (Fig 6), and one-way ANOVA was employed to evaluate the significance of differences among the groups. Results confirmed that the protection efficiency provided by the VP2/4/3 vaccine group was superior to that of the VP2 vaccine group, both of which were significantly higher than the control group (*P* < 0.05), indicating that the virus vaccine group conferred certain protection for chickens.

## Discussion

Infectious bursal disease not only causes the death of chicks, but also destroys the immature B lymphocytes of central immune organ bursa, resulting in suppression of the immune response to various vaccines [18]. Inactivated and live vaccines currently used against IBDV have shortcomings, and therefore, a novel vaccine is urgently needed.

In this study, a baculovirus vaccine was constructed, which combined the advantages of subunit vaccines and DNA vaccines. The baculovirus dual expression system was constructed so that the baculovirus replicated in insect cells and formed virus-like particles, which were then used to infect chicken embryo fibroblast cells. VP2 antigen protein was then synthesized by the host cell transcription system to elicit the immune response and protect chickens against the IBDV virulent strain. The vaccine stimulates the host to produce a long-term immune response, and it can be made into a multivalent vaccine. Additionally, it has a lower cost and is safer, than an attenuated live vaccine.

After immunization, we found that the levels of antibody gradually increased with the vaccination time in chickens with the recombinant baculovirus carrying the *VP2* antigen gene. According to the serum neutralization test results, the neutralizing antibody titer of the VP2 experimental group is only about one-third of the polyprotein VP2/4/3 experimental group after 42 days of immunization. ELISA results showed that the antibody level induced by the VP2 experimental group was lower than that of the VP2/4/3 experimental group, which implied that there were some factors affecting the correct conformation of the VP2 protein that hindered its function in the immune response. In contrast, vaccines constructed based on the *vp2/4/3* gene can elicit higher antibody levels. This was probably due to the guidance of the VP4 protease which helps the VP2 protein to maintain the correct conformational structure. In addition, the VP3 protein could be involved in stabilizing the antigenic determinant site of VP2 as well as increasing the total amount of antigen capture and thereby enhancing the immunogenicity.

Research into subunit vaccines has shown that the major protective antigen of IBD can be successfully expressed by baculovirus; However, the further application of this method is dependent on the suitable immune adjuvant. In addition, many researchers are committed to the study of utilizing the baculovirus vector as a DNA vaccine [19]. DNA vaccines expressed in the host cells can induce humoral and cellular immunity simultaneously, with the same effect as attenuated live vaccines and they can easily be made into a multivalent vaccine.Alpha

Prior to a recent publication [20], there had been no reports on using recombinant baculovirus directly to infect poultry cells, where the dual expression system had been used to prepare the poultry vaccine. Our research group utilized the recombinant baculovirus to develop a novel genetically engineered vaccine that is endowed with the advantages of traditional poultry vaccines and other genetically engineered vaccines. It has the advantages of both recombinant viral vector vaccines and DNA vaccines because of its simulation of natural virus infection and non-replicating features, which will play a significant role in the prevention and treatment of viral diseases. However, the immunization program should further optimized to enhance the immune effect in subsequent trials. Moreover, there are researches we would like to carry out regarding the immune mechanism of buculovirus in the future.

### Conclusions

In this study, recombinant baculoviruses BV-S-ITRs-VP2 and BV-S-ITRs-VP- 2/4/3 were successful constructed and expressed in the chicken embryo fibroblast cells (S1 Table). Results revealed that the neutralizing antibody level induced by the VP2/4/3 vaccine group was higher than that induced by the VP2 vaccine group (S3 Table). The immunized experiments demonstrated that the protection efficiency provided by the VP2/4/3 vaccine group was superior to that of the VP2 vaccine group (S2 and S4 Tables). It was turned out that the recombinant baculoviruses vaccine we prepared conferred good protection for chickens.

## Supporting Information

S1 Table The relative expressed intensity of VP2 protein. (DOC)

S2 Table Antibody levels produced by chickens in each group. (DOC)

S3 Table Neutralizing antibody titer induced by chickens in each group. (DOC)

S4 Table The mean bursa-weight/body-weight index (BBIX). (DOC)
