# Supplementary material for: Construction of Recombinant Baculoviruses Expressing Infectious Bursal Disease Virus Main Protective Antigen and Their Immune Effects on Chickens
Source: PLoS One. 2015 Jul 13;10(7):e0132993. doi: 10.1371/journal.pone.0132993 (PMC4500495; doi:10.1371/journal.pone.0132993)
Supplement: S1 Table — (DOC) [file pone.0132993.s001.doc]

**Supporting Information Table S1. The relative expressed intensity of VP2 protein.**

| **Goup** | **Relative expressed intensity of VP2** |
| --- | --- |
| **BV-S-ITRs-VP2/4/3** | 2.4319±0.0706 |
| **BV-S-ITRs-VP2** | 2.2719±0.1105 |
| ***β*-actin** | 1.0000±0.1002 |
