# Supplementary material for: Construction of Recombinant Baculoviruses Expressing Infectious Bursal Disease Virus Main Protective Antigen and Their Immune Effects on Chickens
Source: PLoS One. 2015 Jul 13;10(7):e0132993. doi: 10.1371/journal.pone.0132993 (PMC4500495; doi:10.1371/journal.pone.0132993)
Supplement: S2 Table — (DOC) [file pone.0132993.s002.doc]

**S2 Table. Antibody levels produced by chickens in each group.**

| **Group**  **Days** | **BV-ITRs-VP2** | **BV-ITRs-VP2/4/3** | **BV-S** | **Vaccine** | **Challenged control** |
| --- | --- | --- | --- | --- | --- |
| **14d** | 0.25±0.014 | 0.253±0.013 | 0.185±0.012 | 0.213±0.012 | 0.211±0.012 |
| **21d** | 0.322±0.013 | 0.445±0.012 | 0.251±0.011 | 0.33±0.012 | 0.25±0.014 |
| **28d** | 0.437±0.015 | 0.632±0.011 | 0.259±0.013 | 0.527±0.014 | 0.243±0.014 |
| **35d** | 0.626±0.013 | 0.941±0.015 | 0.278±0.014 | 0.74±0.015 | 0.267±0.015 |
| **42d** | 0.826±0.014 | 1.231±0.012 | 0.254±0.011 | 1.125±0.013 | 0.249±0.014 |
