# Supplementary material for: Construction of Recombinant Baculoviruses Expressing Infectious Bursal Disease Virus Main Protective Antigen and Their Immune Effects on Chickens
Source: PLoS One. 2015 Jul 13;10(7):e0132993. doi: 10.1371/journal.pone.0132993 (PMC4500495; doi:10.1371/journal.pone.0132993)
Supplement: S3 Table — (DOC) [file pone.0132993.s003.doc]

**S3 Table. Neutralizing antibody titer induced by chickens in each group.**

| **Group**  **Days** | **BV-ITRs-VP2** | **BV-ITRs-VP2/4/3** | **BV-S** | **Vaccine** | **Challenged control** |
| --- | --- | --- | --- | --- | --- |
| **14d** | 5.62±0.47 | 7.32±0.55 | 5.89±0.49 | 8.32±0.62 | 5.26±0.61 |
| **21d** | 41.67±2.21 | 50.00±2.17 | 7.04±0.62 | 23.27±1.67 | 7.94±0.58 |
| **28d** | 132.67±4.99 | 201.08±5.89 | 7.26±0.57 | 86.96±3.10 | 8.12±0.61 |
| **35d** | 250.11±5.89 | 699.01±7.32 | 8.32±0.57 | 322.05±6.03 | 8.25±0.57 |
| **42d** | 887.15±8.32 | 2885.42±11.76 | 8.74±0.70 | 1326.68±8.15 | 8.92±0.56 |
