# Supplementary material for: Construction of Recombinant Baculoviruses Expressing Infectious Bursal Disease Virus Main Protective Antigen and Their Immune Effects on Chickens
Source: PLoS One. 2015 Jul 13;10(7):e0132993. doi: 10.1371/journal.pone.0132993 (PMC4500495; doi:10.1371/journal.pone.0132993)
Supplement: S4 Table — (DOC) [file pone.0132993.s004.doc]

**S4 Table. The mean bursa-weight/body-weight index (BBIX).**

| **Group** | **Mean value of**  **bursa-weight/body-weight index (BBIX)** |
| --- | --- |
| **BV-S-CMV-VP2** | 3.45±0.14 |
| **BV-S-CMV-VP2/4/3** | 4.07±0.19 |
| **BV-S-CMV** | 3.03±0.18 |
| **Vaccine group** | 3.13±0.27 |
| **Challenged control group** | 2.86±0.21 |
